# The Effects of Acute Moderate and High Intensity Exercise on Memory

**DOI:** 10.3389/fpsyg.2020.01716

**Published:** 2020-07-14

**Authors:** David Marchant, Sophie Hampson, Lucy Finnigan, Kelly Marrin, Craig Thorley

**Affiliations:** ^1^Psychology of Sport, Exercise and Movement Research Group, Department of Sport and Physical Activity, Edge Hill University, Ormskirk, United Kingdom; ^2^Manchester University NHS Foundation Trust, Manchester, United Kingdom; ^3^School of Sport and Exercise Sciences, Liverpool John Moores University, Liverpool, United Kingdom; ^4^Department of Psychology, James Cook University, Townsville, QLD, Australia

**Keywords:** acute exercise, exercise intensity, cognition, recall, recognition, false memory

## Abstract

Acute cardiovascular exercise can enhance correct remembering but its impact upon false remembering is less clear. In two experiments, we investigated the effect of acute bouts of exercise on correct and false remembering using the Deese–Roediger–McDermott (DRM) memory test. In Experiment 1, healthy adults completed quiet rest or moderate intensity cycling prior to the memory test. In Experiment 2, a similar sample completed moderate intensity running, high intensity sprints, or a period of quiet rest prior to the memory test. In Experiment 1, acute moderate intensity exercise increased short-term correct, but not false, recall. Experiment 2 replicated these findings but also found an acute bout of high intensity exercise had no impact upon either type of short-term recall. Acute moderate intensity exercise, but not acute high intensity exercise, can improve short-term correct recall without an accompanying increase in false recall potentially through processing of contextually specific information during encoding.

## Introduction

Acute bouts of cardiovascular exercise provide moderate short-term post-exercise enhancements to several cognitive functions, including the speed of mental processing, attention, and executive function (see Chang et al., 2012; McMorris and Hale, 2012). These beneficial effects are associated with increases in arousal and available cognitive resources (e.g., Hillman et al., 2003), which are also proposed to be critical variables in efficient memory functioning. The influence of acute exercise on memory may depend on the temporal relation between the exercise bout, information encoding (the initial perceiving and learning of information), consolidation (memory trace stabilization into long-term memory formation after initial encoding) and subsequent recall (the retrieval of stored information). When exercise occurs immediately before a memory encoding task, there are moderate enhancements to the volume of studied information correctly remembered, whereas exercising during encoding appears to impair encoding (see Loprinzi et al., 2019, for a meta-analysis). Pre-encoding exercise is observed to be more beneficial than post-encoding exercise (Labban and Etnier, 2011), whilst concurrent exercise and encoding may impair subsequent recall (Soga et al., 2017). Furthermore, study characterizes in terms of exercise (intensity, duration), participants (age, fitness) and memory tasks (working memory, episodic memory, prospective memory) can moderate the potential for beneficial effects (Loprinzi, 2018). The majority of work has focused in correct recall, whilst little is known regarding acute cardiovascular exercise prior to either a short-term or long-term memory test can influence false remembering (memory of information that was not present at encoding).

Acute bouts of cardiovascular exercise, relative to rest, can enhance both short and long term explicit, declarative memory (memory that can be consciously recalled) of previously observed word lists. For example, enhancements have been observed after participants engaged in 10 min of brisk walking (e.g., English nouns: Salas et al., 2011), 40 min of moderate intensity aerobic cycling (e.g., English nouns: Coles and Tomporowski, 2008), six min of high intensity anaerobic sprints (e.g., Novel vocabulary: Winter et al., 2007), and 30 min of treadmill running above lactate threshold (e.g., Rey Auditory Verbal Learning Test: Etnier et al., 2016). In reviewing these patterns, when considering the type of memory task employed, Loprinzi (2018) suggests that whilst acute high-intensity exercise pre-memory task may impair working memory it can benefit episodic memory whilst high-intensity exercise post-encoding may not benefit long-term memory performance. These exercise-induced arousal memory benefits are thought to be associated with increased levels of brain-derived neurotrophic factor (BDNF) and catecholamines (dopamine, epinephrine, norepinephrine) (e.g., Winter et al., 2007).

The benefits of acute bouts of exercise on remembering may be of limited use if they are accompanied by increases in false remembering (e.g., recollecting events that did not happen or incorrectly recollecting events that did happen). To date, the impact of an acute bout of exercise on false remembering (as assessed using the DRM protocol) has only received limited examination. Green and Loprinzi (2018) found 15 min of self-paced brisk treadmill walking had no effect on correct or false declarative recall. Whilst Siddiqui and Loprinzi (2018) found that 20 min of brisk treadmill walking benefited accurate recall, with no differences in false recall. In contrast, Dilley et al. (2019) found 15-min high-intensity treadmill running (80%HRR) pre-encoding significantly increased correct recall over moderate intensity (50%HRR) exercise, the latter also enhanced memory over rest. False recall was not significantly impacted by exercise intensity, but the authors tentatively suggest their data indicates higher-intensity exercise may increase false recall. As such, the relationship between acute exercise pre-encoding warrants further consideration.

The Deese/Roediger-McDermott (DRM) paradigm (Roediger and McDermott, 1995) is widely used to induce false memories (see Gallo, 2010, for a review). Participants study lists of words (e.g., bed, dream, wake, snore, etc.) that are semantically associated with a non-presented critical lure word (e.g., sleep). In later assessment, participants frequently falsely recall and falsely recognize these critical lures as a previously studied word with high confidence. The Activation-Monitoring Theory (AMT, Roediger et al., 2001) and the Fuzzy Trace Theory (FTT, Brainerd and Reyna, 2002) explain why DRM word lists induce false memories. The AMT posits that, during encoding, the studied words (either consciously or unconsciously) activate the semantically associated critical lure. During subsequent memory tests, participants generate words based on their semantic activation at encoding. As the critical lure was activated at encoding, they commit a source-monitoring error and class it as a presented word (instead of an internally generated non-studied word). The FTT posits that participants generate two parallel memory traces (changes in the nervous system representing information) for studied words at encoding: verbatim traces (precise memory representations) and gist traces (vague meaning-based memory representations). The critical lures are strongly associated with gist of studied lists and are incorrectly presumed to have been studied. The AMT and FTT accounts are not mutually exclusive and both implicate the semantic association between studied lists and the non-studied critical lures in false remembering.

Currently, Loprinzi and colleagues (e.g., Siddiqui and Loprinzi, 2018) have tentatively suggested that moderate-intensity exercise may benefit accurate whilst reducing false declarative recall, whilst high-intensity exercise may elevate both accurate and false recall (Dilley et al., 2019). Consistent with this possibility, there is converging evidence from two distinct lines of research that self-reported arousal (admittedly, from other sources) can increase false remembering. Firstly, caffeine-induced arousal increased false recall of critical lures on memory tests (e.g., Mahoney et al., 2012). The second line of research demonstrates that high pre-encoding emotion-induced arousal can increase false remembering (e.g., Corson and Verrier, 2007). Taken together, self-reported arousal is often associated with increases in short-term memory false recall. Mahoney et al. (2012) and Corson and Verrier (2007) suggest that elevated arousal increases relational processing rather than item-specific processing at encoding in explain these effects. Item-specific processing focusses attention on individual studied items and how they are distinct from each other, whereas relational processing focusses participants’ attention on the commonalities amongst studied items. Roediger et al. (2001) suggest that relational processing intensifies the spread of activation to the critical lures, which are more likely to be falsely remembered. These findings suggest some forms of arousal can increase false remembering and it is of interest to know whether this effect generalizes when the arousal is exercise-induced. For example, exercise-induced arousal (proposed to be associated with increases in neurotransmitters norepinephrine and dopamine) benefits speed of cognitive processing (McMorris and Hale, 2012).

The present within-subjects experiments examined the impact of a single acute bout of exercise on explicit, declarative short-term memory. Specifically, the effects of acute exercise undertaken immediately prior to encoding on subsequent short-term declarative correct and false remembering, as assessed via a DRM memory test. As exercise intensity, and therefore the degree of probable arousal, influences correct remembering, exercise intensity was manipulated here to see if it influences false remembering. More specifically, Experiment 1 examined whether engaging in an acute bout of moderate intensity aerobic exercise prior to encoding, relative to rest, impacts upon short-term declarative correct and false recall/recognition. Experiment 2 expanded this by examining whether engaging in acute moderate intensity aerobic exercise or high intensity anaerobic exercise prior to encoding, relative to rest, impacts upon short-term declarative correct and false recall/recognition. In both studies, it was anticipated that an acute bout of exercise prior to encoding would enhance short-term correct remembering. Despite previous findings (e.g., Green and Loprinzi, 2018), it is also tentatively predicted than an acute bout of exercise will elevate short-term false remembering as other forms of arousal (i.e., caffeine-induced, emotion-induced) are associated with increased false remembering (e.g., Corson and Verrier, 2007; Mahoney et al., 2012).

## Experiment 1

### Methods

#### Participants

Twenty six healthy and regularly physically active (>3 aerobic exercise sessions on three days per week for at least 30 min per session, >2 years of regular exercise participation) young adults (19M, 7F, Mage = 22.19 + 3.15 years) participated in the study after giving written informed consent. An *a priori* power analysis (using G^∗^Power 3.1) with an α level of 5%, medium-to-large effect size (*d* = 0.6), and a power of 80%, based on effect sizes reported in previous within-subjects work (e.g., Etnier et al., 2016, *N* = 16; Labban and Etnier, 2018, *N* = 15) indicated that at least 19 participants would be required. All spoke English as their first language, had normal or corrected to normal vision, had no history of mood disorders, and were not taking any medication that would affect cognition. Participants were asked to refrain from strenuous physical exercise on the day of testing, to avoid caffeine and alcohol intake for 24 h prior to testing, to arrive appropriately hydrated, and to have not eaten for a minimum of 3 h.

### Experimental Design

In a within-subjects design, participants completed two activities (rest or exercise) in a counterbalanced order. In the rest condition, participants were seated for 30 min prior to a DRM memory test. In the exercise condition, participants completed 30 min of moderate intensity exercise prior to a DRM memory test.

### Activities and Measures

#### Rest Protocol

Participants were seated alone at a table in a quiet room and given the opportunity to read popular magazines for 30 min. This has been shown to be an acceptable rest activity the avoids boredom and does not impact memory function (Blough and Loprinzi, 2019). Participants were monitored to ensure they did not fall asleep or stand up and move around. Ratings of perceived exertion (RPE; individual’s perceptions of exercise intensity) and affective valence (pleasure-displeasure) were recorded every two min.

#### Exercise Protocol

On a cycle ergometer (Monark, Model: 824E, Country: Sweden) participants completed a 5-min self-paced warm-up, followed by 30 min of moderate intensity cycling. Participants were asked to self-regulate a level of perceived exertion between *somewhat hard* and *hard* (within the 13–15 range on the Borg RPE scale), and were free to adjust their cadence to maintain their RPE within the target range. A reminder of the target RPE range was continuously in sight, and the researcher reminded participants every 2 min during exercise (offset from RPE measurement). RPE is a validated and practical perceptual method of directing self-selected exercise intensity, and this protocol has previously been used (e.g., Labban and Etnier, 2011) to ensure participants exercise at a moderate intensity below the ventilatory threshold. RPE, affective valence (pleasure-displeasure), and heart rate (HR) were recorded every two min. Water was available *ad libitum* throughout exercise.

#### Ratings of Perceived Exertion (RPE)

The Borg RPE scale (Borg, 1998) ranging from 6 (no exertion at all) to 20 (maximal exertion) was used to assess subjective interpretations of effort during exercise. The RPE scale is a widely accepted and validated method for estimating perceptions of exercise intensity, demonstrating high correlations (r = 0.80–0.90) between RPE and HR (Borg, 1998).

#### Affect

The Feeling Scale (FS; Hardy and Rejeski, 1989) is a single-item measure of pleasure and displeasure (how are you feeling right now?), 11-point bipolar rating scale ranging from + 5 (I feel very good), zero (neutral), to –5 (I feel very bad).

#### Heart Rate (HR)

Participants’ HR was continuously monitored via a Polar Rate Monitor (Model A1; Polar Electro, Kempele, Finland) and recorded every 2 min.

#### Memory Task

The DRM paradigm (Roediger and McDermott, 1995) consisted of twelve lists of 15 word rated by Stadler et al. (1999) as producing high levels of false recognition (critical lures: Window, Doctor, Smoke, Anger, Cup, Slow, Sleep, Sweet, Rough, Soft, Cold, River). Participants were provided with standardized instructions on the task by the researcher, and this was repeated on the introductory computer screens. Two sets of 6 lists were assigned to each condition in counterbalanced order. Words were presented sequentially on a computer screen at a rate of 2s per word with a 1 s interval. Immediately after list, participants undertook a 1-min free recall test under the instruction to write as many words as possible using pen and paper. The number of correctly recalled old items (studied words), falsely recalled lures, and intrusions was totaled across the six lists. After the final free recall test, participants completed a 36-word recognition test similar to Knott and Thorley (2014). This test contained the six critical lures, 18 previously studied words (from positions one, five, and ten in each studied list), and 12 non-studied new words not semantically associated with the studied words or critical lures. On a paper response sheet, participants indicated whether the words were old (the word was previously studied) or new (the word was not previously studied). Old words were then rated as remember (recollect some contextual detail of seeing the word during encoding), know (recognized the word based on familiarity but had no recollection of any contextual information), or guess. Correct recognition of studied words, false recognition of critical lures, and incorrect recognition of non-studied new words (i.e., not critical lures) were calculated as proportions.

### Procedure

Participants completed each condition activity (rest or exercise) individually on separate days at the same time of day in the same well-controlled laboratory setting led by the same researcher. There was a seven-day rest period between each session to control for factors that may affect memory performance (e.g., carry over effects). In the first session, participants read and signed an informed consent sheet, completed exercise readiness and health screening, and were familiarized with all equipment and testing procedures. On each day, participants first undertook the condition activity and then immediately completed the DRM memory tasks whilst seated in front of a computer in a distraction free testing booth situated next to the activity area. Upon completion of the activity, transition to the testing booth, reinstruction and commencing the memory task took approximately 2 min. A researcher was always present in the laboratory with the participant, and they provided the same instructions and monitoring to all participants. Following the completion of the final condition, all participants were debriefed about the aim of the experiment.

### Statistical Analysis

Recall and recognition performance was analyzed with MANOVA followed by univariate ANOVAs using SPSS (version 22.0; International Business Machines Corp., Armonk, NY, United States), considering Condition (Rest vs. Exercise) as the within-subjects factor and word type (Recall: Critical Lure, Correct Recall, Other Error. Recognition: Critical Lure, Studied Word, New Word) as the dependent variables. If Mauchly’s Test indicated sphericity was violated, a Greenhouse-Geisser correction was employed (εGG is reported in such cases). Partial eta-squared (ηp2) proportion of variance effect size was calculated and described with Cohen’s (1988) cut-off points (i.e., small = 0.0099; medium = 0.0588; large = 0.1379).

## Results

### Exercise Intensity

To check that the self-selected approach to targeting exercise intensity was successful, HR, RPE and Affect are assessed. The mean HR during exercise was 136 bpm (±17.34), indicating exercise was undertaken at an average of 68.40% of HRmax. The mean RPE reported during exercise was 12.65 (±1.23). Taken together, exercise was at a self-selected moderate intensity (Garber et al., 2011). Average affective responses were significantly more positive in the rest condition (3.38, ±1.81) compared to the exercise condition (1.71 ± 1.12), t (25) = 4.08, *p* < 0.001.

### Short-Term Free Recall

The results showed a significant multivariate effect of Condition, λ = 0.70, *F*(3, 23) = 3.28, *p* = 0.038, η_*p*_^2^ = 0.30. The follow-up univariate analyses confirmed that the difference between Rest and Exercise was significant for Correct Recall (42.69 ± 9.21 vs. 39.35 ± 7.03), *F*(1, 25) = 4.81, MSE = 145.56, *p* = 0.038, η_*p*_^2^ = 0.16. Contrary to our tentative prediction, there was no significant difference in the number of critical lures falsely recalled in the exercise (2.69 ± 1.52) and rest conditions (2.08 ± 1.44), *F*(1, 25) = 3.32, MSE = 4.92, *p* = 0.08, η_*p*_^2^ = 0.12. There was no significant main effect of condition on other recall errors (2.88 ± 3.83 vs. 1.81 ± 1.90), *F*(1, 25) = 0.89, MSE = 3.25, *p* = 0.35, η_*p*_^2^ = 0.03 (see Table 1).

**TABLE 1:** Mean (SD) number of studied words correctly recalled (max = 90), critical lures falsely recalled (max = 6), and non-studied new words incorrectly recalled following two activities (rest or moderate intensity exercise) in Experiment 1.

Activity	Studied words	Critical lures	Non-studied new words
Rest	39.35 (7.03)	2.08 (1.44)	2.89 (3.82)
Exercise	42.69 (9.21)^a^	2.69 (1.52)	1.81 (1.90)

*^*a*^Significantly different from rest.*

### Recognition Memory

No significant multivariate effect of Condition on the proportion of words was recognized, λ = 0.99, *F*(1, 25) = 0.17, *p* = 0.68, η_*p*_^2^ = 0.01, nor Condition x Word Type interaction λ = 0.99, *F*(1, 24) = 0.03, *p* = 0.97, η_*p*_^2^ = 0.02 (see Table 2). Similarly, a MANOVA with Condition (Rest vs. Exercise) and Word Type (Studied vs. Critical Lure vs. New) as within-subjects factor, and judgment type (Remember, Know, Guess) as the dependent variables showed no significant multivariate effect of Condition on the proportion of words were recognized, λ = 0.97, *F*(3, 23) = 0.24, *p* = 0.87, η_*p*_^2^ = 0.03, nor Condition x Word Type interaction λ = 0.91, *F*(6, 20) = 0.34, *p* = 0.91, η_*p*_^2^ = 0.09. An equivalent, but large, proportion of studied words were recognized and critical lures falsely recognized in both conditions, whilst participants recognized few non-studied new words across conditions. Therefore contrary to expectations, exercise did not impact upon true and false recognition.

**TABLE 2:** Mean (SD) proportion of studied words correctly recognized, critical lures falsely recognized, and non-studied new items incorrectly recognized following each activity in Experiment 1.

		Rest	Moderate exercise
Studied Words	Old (*Correct*)	0.78 (0.12)	0.77 (0.19)
	Remember	0.38 (0.27)	0.44 (0.32)
	Know	0.27 (0.26)	0.25 (0.28)
	Guess	0.12 (0.17)	0.07 (0.06)
Critical Lures	Old (*Incorrect*)	0.87 (0.17)	0.87 (0.20)
	Remember	0.47 (0.34)	0.47 (0.34)
	Know	0.31 (0.28)	0.29 (0.30)
	Guess	0.09 (0.12)	0.11 (0.14)
New Words	Old (*Incorrect*)	0.19 (0.23)	0.17 (0.25)
	Remember	0.04 (0.07)	0.05 (0.12)
	Know	0.04 (0.09)	0.02 (0.07)
	Guess	0.11 (0.18)	0.10 (0.21)

## Discussion

Experiment 1 demonstrated that acute moderate intensity aerobic exercise, relative to a period of rest, prior to a DRM memory task increased short-term correct recall. This is consistent with past research showing that exercise improves the ability to retain and recall information in short-term memory (Loprinzi et al., 2019). Contrary to expectations, participants’ long-term correct recognition of studied words was equivalent regardless of whether they were tested after exercise or rest. Short-term correct recall and long-term correct recognition are, therefore, differentially impacted upon by exercise in this study. In a novel comparison, we also found that moderate intensity aerobic exercise, relative to rest, has no impact upon number of critical lures (or other non-studied words) falsely recalled during a short-term memory test or falsely recognized during a long-term memory test. Supporting initial observations (Siddiqui and Loprinzi, 2018) this suggests exercise-induced arousal, unlike other forms of arousal (e.g., caffeine-induced, mood-induced) does not increase false remembering. It remains to be determined whether intensity can also influence false remembering. Furthermore, as the recognition test took place several min after the exercise had finished, it is unclear whether participants arousal levels had deteriorated in the exercise condition, so they were on par with those in the rest condition. If so, that could account for the null results. Experiment 2 therefore measures participants’ arousal levels prior to and after each memory test.

## Experiment 2

Experiment 2 compared the impact of rest, moderate intensity aerobic exercise, and high intensity anaerobic exercise on correct and false remembering as assessed via a short-term memory free recall test and a long-term memory recognition test. Consistent with several past studies (e.g., Winter et al., 2007), we used running protocols to elicit moderate and high intensity exercise. In methodological improvements to Experiment 1, we tailored the intensity in line with participants’ individual fitness level.

### Methods

#### Participants

Twenty-five healthy, normally functioning, and physically active males volunteered for the study (M_*age*_ = 25.84 ± 6.46 years). Each had considerable prior experience of high intensity treadmill running. The inclusion, exclusion, and screening procedures were identical to Experiment 1. All participants were naïve to the aims of the study. Participants refrained from strenuous physical exercise on testing days, avoided caffeine and alcohol intake for 24 h prior to testing, and arrived appropriately hydrated, and having not eaten for a minimum of 3 h.

### Experimental Design

In a within-subjects design modeled on that of Winter et al. (2007), participants completed three activities (rest, moderate intensity aerobic exercise, high intensity anaerobic exercise) in a counterbalanced order. In the rest condition participants rested for 40 min prior to a DRM memory test, in the moderate intensity exercise condition participants completed 40 min of steady state running prior to a DRM memory test, and in the high intensity exercise condition participants completed 2 × 3 min of sprints prior to a DRM memory test.

### Activities and Measures

#### Rest Protocol

Similar to Study 1, participants sat quietly in the laboratory for 40 min and were given popular magazines to read, and were monitored throughout.

#### Exercise Protocols

Running was undertaken on a h/p/cosmos pulsar 3p treadmill (h/p/cosmos sports & medical GmbH). In the first testing session, participants’ baseline fitness levels were assessed. In that session, participants completed a treadmill based graded exercise test to determine their VO2peak and HRmax. Oxygen uptake (VO2) was measured using a METAMAX cardiopulmonary exercise testing system (Cortex Biophysik GmbH) with breathing mask, pre-calibrated according to the manufacturer’s instructions. A computerized indirect calorimetry system collected 30-s averages for oxygen uptake (VO2) and respiratory exchange ratio (RER). ***Moderate intensity exercise***: Participants completed 40 min of continuous moderate intensity running consisting of a 5-min warm-up (work rate at 30% of VO2peak), followed by 35 min of exercise at 60% of VVO2peak (Velocity at Vo2 Peak). ***High intensity exercise***: this condition aimed to achieve a very high intensity and high blood-lactate concentration (10 mmol/l or above) while limiting the total exercise duration, fatigue and dehydration. Participants completed a 5-min warm-up (30% of VO2peak), and then completed two incremental maximal efforts (3 min each), separated by 2 min passive recovery. In line with Winter et al. (2007) running protocol description, the treadmill speed started at 8 km/h, and increased every 10s by 2 km/h, until volitional exhaustion.

#### Heart Rate (HR), Affective Valence, Ratings of Perceived Exertion

These were measured in an identical manner to Experiment 1.

#### Arousal

Participants reported subjective arousal levels using 20-item Activation-Deactivation Adjective Checklist (ADCL), to generate four subscales; energy, tiredness, tension, and calmness. Participants rate affect adjectives on a four-point scale: definitely feel, slightly feel, cannot decide, definitely do not feel. The ADCL has acceptable reliability and validity (Thayer, 1989).

#### Blood-Lactate

Blood-lactate levels for moderate and high intensity running exercise was predicted to be above 10 mmol/l or below 2 mmol/l, respectively (Spurway, 1992). Blood-lactate was measured from fingertip capillary blood samples using an automated analyzer (Analox GM7 enzymatic metabolite analyzer, Analox instruments USA, Lunenburg, MA) immediately post-exercise and between recall and recognition tasks.

#### Memory Task

The DRM paradigm followed the same protocols from Experiment 1. Eighteen DRM lists of 15 words (critical lures: Window, Doctor, Smoke, Anger, Cup, Slow, Sleep, Sweet, Rough, Soft, Cold, River, Smell, Chair, Needle, City, Mountain, Spider) were divided into three sets of six and their assignment to each condition was counterbalanced. The recognition tests were constructed in a similar manner to Experiment 1.

### Procedure

Participants individually attended four sessions (an initial screening and baseline fitness test, followed by the three activity conditions) on separate days at the same time of day (to control for diurnal variation) in the same laboratory. A seven-day rest period between each session controlled for fatigue and memory carry over effects. In the first session, participants read and signed an informed consent sheet, completed a pre-exercise health screening, the fitness test and were introduced to the memory task. In the second, third, and fourth sessions, the order of the three conditions were counterbalanced. To avoid dehydration, water was available *ad libitum* throughout exercise and participant were instructed to arrive hydrated. The same researcher provided the same instructions and monitored the participant during all activities and testing. Participants’ RPE, affect, and HR were taken every 2 min during moderate intensity exercise and rest, and in the last min of each activity (sprint and recovery) of the high intensity condition. Immediately after each activity, a DRM memory test was completed in a distraction free testing booth situated next to the activity area. Upon completion of the activity, transition from the activity to the testing booth, reinstruction and commencing the memory task took approximately 2 min. A blood-lactate sample was taken and the ADCL was completed four times per experimental session: (1) before undertaking the activity (2) immediately prior to the free recall test, (3) immediately prior to the recognition test, and (4) after the recognition test. Following the final testing session, participants were debriefed regarding the aims of the experiment.

### Statistical Analysis

Pre-activity blood-lactate, pre-activity arousal, average RPE, FS ratings, and HR were compared using one-way repeated measures ANOVAs. Post-activity blood-lactate was analyzed using two-way (Time × Activity) repeated-measures ANOVA. ADCL subscale ratings were analyzed using a MANOVA considering Condition (Rest vs. Moderate Exercise vs. High Intensity Exercise) and Time (Pre-Activity, Pre-free recall test, Pre-recognition test, and post-recognition test) as the within-subjects factor, and ADCL subscale (Energy, Calmness, Tiredness, Tension) as dependent variables. Mean number of studied words correctly recalled, critical lures falsely recalled, and non-studied new words incorrectly recalled in the three activity conditions was analyzed with a MANOVA followed by univariate ANOVAs, considering Condition (Rest vs. Moderate Exercise vs. High Intensity Exercise) as the within-subjects factor, and recall type (correct recall, critical lure, and other errors) as dependent variables. For the assessment of recognition, a MANOVA with Condition (Rest vs. Moderate Intensity Exercise vs. High Intensity Exercise) as the within-subjects factor and word type (Critical Lure, Studied Word, New Word) as the dependent variables was employed. If Mauchly’s Test indicated sphericity was violated, a Greenhouse-Geisser correction was employed (εGG is reported in such cases). Partial eta-squared (η_*p*_^2^) is reported as a measure of effect-size.

## Results

### Baseline Arousal and Blood-Lactate

There were no significant multivariate effect of Condition on participants’ baseline arousal levels, assessed via the four ADCL subscales, prior to each of the three activities, λ = 0.91, *F*(8, 90) = 0.53, *p* = 0.83, η_*p*_^2^ = 0.05. Similarly, there was no significant difference in their baseline blood-lactate levels prior to each activity, *F*(2, 48) = 2.63, *p* = 0.08, η_*p*_^2^ = 0.10 (see Table 3).

**TABLE 3:** Blood Lactate and Arousal responses (M ± SD) before and after the three activities (rest, moderate intensity exercise, high intensity exercise) in Experiment 2.

Measure	Time	Rest	Moderate exercise	Intense exercise
Blood Lactate mmol/l	Pre-activity	1.00 (0.41)	1.13 (0.42)	1.27 (0.45)
	Post-activity	1.08 (0.42)	1.78 (0.76)	6.20 (0.55)
	Post-Recall	1.01 (0.36)	1.74 (0.58)	6.22 (1.48)
	Post-recognition	0.96 (0.29)	1.51 (0.43)	5.60 (1.35)
Energy	Pre-activity	9.84 (4.09)	11.00 (4.93)	11.44 (5.08)
	Post-activity	7.88 (4.39)	13.40 (4.74)	15.36 (4.92)
	Post-Recall	10.12 (4.60)	10.80 (4.71)	10.00 (5.33)
	Post-recognition	9.96 (4.31)	9.96 (4.78)	10.36 (5.31)
Calmness	Pre-activity	14.08 (3.55)	13.60 (5.43)	13.04 (4.06)
	Post-activity	15.80 (5.26)	10.24 (3.32)	8.56 (3.92)
	Post-Recall	14.88 (5.87)	13.08 (5.34)	13.56 (3.92)
	Post-recognition	14.24 (4.87)	13.28 (3.92)	13.64 (4.21)
Tiredness	Pre-activity	9.12 (5.59)	8.52 (4.50)	8.60 (3.98)
	Post-activity	10.28 (5.73)	6.24 (3.36)	7.48 (6.47)
	Post-Recall	9.64 (5.18)	8.16 (4.90)	9.88 (5.15)
	Post-recognition	9.76 (5.44)	8.32 (4.71)	9.32 (5.48)
Tension	Pre-activity	7.44 (3.34)	7.76 (3.11)	7.68 (3.48)
	Post-activity	7.28 (2.85)	8.68 (3.04)	8.96 (2.26)
	Post-Recall	7.80 (3.23)	7.84 (3.47)	8.00 (2.69)
	Post-recognition	7.12 (2.99)	7.64 (3.60)	7.36 (2.38)

### End of Activity Exertion, Heart Rate, and Affect

RPE was significantly different in the final minute of each condition, *F*(1.33, 31.96) = 108.57, *p* < 0.001, η_*p*_^2^ = 0.82, εGG = 0.67. As would be expected, participants had significantly higher average RPE during high intensity exercise (16.36 ± 3.32) compared to moderate intensity exercise (12.68 ± 2.39; *p* < 0.001) and rest (6.12 ± 0.44; *p* = 0.001) conditions, and these latter conditions were also statistically different (*p* < 0.001). HR was also significantly different in the final minute of each condition, *F*(2, 48) = 632.99, *p* < 0.001, η_*p*_^2^ = 0.96, with high intensity exercise (170bpm ± 12.55, 88.04% HRmax) producing significantly higher average HR than both moderate intensity exercise (154.08 bpm ± 15.52, 79.43% HRmax; *p* < 0.001) and rest (66.12 bpm ± 7.34, 34.03% HRmax; *p* < 0.001). The latter two activities HR were also significantly different (*p* < 0.001). Affect was significantly different between conditions, *F*(2, 48) = 17.48, *p* < 0.001, η_*p*_^2^ = 0.42, with high intensity exercise inducing less positive affect (0.24 ± 2.52) than moderate intensity exercise (2.40 ± 1.76; *p* < 0.001) and rest (3.04 ± 1.67; *p* < 0.001). End of activity affect was equivalent for the latter two activities (*p* = 0.59). Finally, self-reported arousal in the final minute of each activity condition was significantly different, *F*(2, 48) = 32.91, *p* < 0.001, η_*p*_^2^ = 0.58. High intensity exercise (4.24 ± 1.56) induced average arousal levels significantly higher than moderate intensity exercise (3.32 ± 1.38; *p* = 0.02) and rest (1.92 ± 1.19; *p* < 0.001), which were themselves different (*p* < 0.001) (see Table 4). Combined, the above confirm that participants felt more exerted, aroused, and less pleasurable, in the final minute of the high intensity exercise than the moderate intensity exercise and period of rest. Moreover, they felt more exerted and aroused, but no less pleasurable, toward the end of the moderate intensity exercise than the period of rest.

**TABLE 4:** During-activity RPE, HR (bpm), Affect and arousal (Mean ± SD) from final section of the three activities (rest, moderate intensity exercise, high intensity exercise) in Experiment 2.

Activity	RPE	HR (bpm)	Affect	Arousal
Rest	6.12 (0.44)^b,c^	66.12 (7.34)^b,c^	3.04 (1.67)^b,c^	1.92 (1.19)^b,c^
Moderate Exercise	12.68 (2.39)^a,c^	154.08 (15.52)^a,c^	2.40 (1.76)^a,c^	3.32 (1.38)^a,c^
Intense Exercise	16.36 (3.32)^a,b^	170 12.55)^a,b^	0.24 (2.52)^a,b^	4.24 (1.56)^a,b^

*^*a*^Significantly different from rest, ^*b*^significantly different from moderate intensity exercise, ^*c*^significantly different from high intensity exercise.*

### Post-activity Blood-Lactate

Our two-way analyses of post-activity blood-lactate levels revealed a significant main effect of Activity, *F*(1.33, 31.94) = 283.78, *p* < 0.001, η_*p*_^2^ = 0.92, εGG = 0.67. *Post hoc* analysis revealed that blood-lactate levels were significantly higher after high intensity exercise (6.01 ± 1.25 mmol/l) compared to moderate intensity exercise (1.68 ± 0.5 mmol/l; *p* < 0.001) and rest (1.02 ± 0.3 mmol/l; *p* < 0.001). Moreover, moderate intensity exercise resulted in higher post-exercise blood-lactate levels than rest (*p* < 0.001). There was also a main effect of Time, *F*(1.24, 29.79) = 6.81, *p* = 0.002, η_*p*_^2^ = 0.22, εGG = 0.62, indicating decreasing BL post-exercise. *Post hoc* analyses showed that blood-lactate levels were lowest Post-Recognition (2.69 ± 2.5 mmol/l) compared to Post-Exercise (3.02 ± 3.0 mmol/l; *p* = 0.03) and Post-Recall (2.99 ± 2.5 mmol/l; *p* < 0.001), which were themselves not different (*p* = 0.12). There was no Activity x Time interaction, *F*(1.61, 38.74) = 1.55, *p* = 0.15, η_*p*_^2^ = 0.07, εGG = 0.40. Together, this indicates exercise increased peripheral BL concentrations, and intense exercise resulted in the greatest elevation indicative of greater workload (see Table 3). However, whilst moderate intensity BL concentrations are in line with expectations, the intense anaerobic exercise did not reach the intended threshold (e.g., lactate levels above 10 mmol/l, Spurway, 1992).

### Post-activity Arousal

The results showed a significant multivariate Condition x Time interaction, λ = 0.58, *F*(24, 493) = 3.45, *p* < 0.001, η_*p*_^2^ = 0.13. The follow-up univariate analyses confirmed significant Condition x Time interactions for Energy, *F*(4.55, 109.17) = 13.67, MSE = 108.74, *p* < 0.001, η_*p*_^2^ = 0.36, εGG = 0.76, Calmness, *F*(4.02, 96.46) = 6.54, MSE = 100.65, *p* < 0.001, η_*p*_^2^ = 0.21, εGG = 0.67, but not Tiredness, *F*(3.42, 81.97) = 1.93, MSE = 32.56, *p* = 0.12, η_*p*_^2^ = 0.07, εGG = 0.57, or Tension, *F*(4.33, 103.96) = 1.14, MSE = 5.40, *p* = 0.34, η_*p*_^2^ = 0.05, εGG = 0.72. Energy levels increased post-activity for both exercise conditions when compared to rest (which had reduced from baseline), these returned to comparable levels post-recall and recognition. There were lower calmness levels post-activity for both exercise conditions compared to rest (which increased from baseline), with the lowest calmness post-intense exercise. These returned to comparable levels post-recall and recognition, albeit with calmness remaining high for the rest condition. For Tiredness, there was a significant multivariate, λ = 0.63, *F*(8, 90) = 2.89, *p* < 0.01, η_*p*_^2^ = 0.20, and univariate, *F*(2, 48) = 3.30, MSE = 89.44, *p* < 0.05, η_*p*_^2^ = 0.12, effect of Condition. Bonferroni-adjusted pairwise comparisons revealed that Tiredness was significantly lower in the Moderate Intensity Exercise condition (7.81 ± 3.62) than Rest (9.70 ± 4.74, p = 0.02), but not High-Intensity Exercise (8.82 ± 4.63, *p* = 0.59). The latter two conditions were also not significantly different (*p* = 0.84). Taken together, both forms of exercise resulted comparable reductions in calmness and increases in energy post-activity, which returned to baseline post- recognition (see Table 3). Tiredness was lowest during all phases post-moderate intensity exercise. There was a trend for larger effects of intense exercise on energy and calmness. Rest had a smaller, yet opposite effect on these characteristics.

### Short-Term Free Recall

Table 5 shows mean number of studied words correctly recalled, critical lures falsely recalled, and non-studied new words incorrectly recalled in the three activity conditions. Results showed a significant multivariate effect of Condition, λ = 0.76, *F*(6, 92) = 2.28, *p* = 0.042, η_*p*_^2^ = 0.13. The follow-up univariate analyses confirmed a significant main effect of condition for Correct Recall, *F*(2, 48) = 4.96, MSE = 158.01, *p* = 0.011, η_*p*_^2^ = 0.17. As expected, Bonferroni-adjusted pairwise comparisons revealed that more studied words were correctly recalled after moderate intensity (47.20 + 8.36) exercise than rest (42.20 ± 8.36, *p* = 0.005). Contrary to expectations, there were no significant differences in the correct recall after moderate intensity and high intensity exercise (45.16 ± 9.87, *p* = 0.52) or after high intensity exercise and rest (*p* = 0.39). Importantly, the activity engaged in had no impact upon the number of critical lures falsely recalled, *F*(2, 48) = 0.16, MSE = 0.28, *p* = 0.85, η_*p*_^2^ = 0.01, or the number of non-studied new words incorrectly recalled *F*(1.22, 46.33) = 1.82, MSE = 37.92, *p* = 0.19, η_*p*_^2^ = 0.07 (Greenhouse-Geisser corrections applied to the latter main effect).

**TABLE 5:** Mean (SD) number of studied words correctly recalled (max = 90), critical lures falsely recalled (max = 6), and non-studied new words (not critical lures) incorrectly recalled after the three activities in Experiment 2.

Activity	Studied words	Critical lures	Non-studied words
Rest	42.20 (8.36)	2.68 (1.65)	1.52 (1.66)
Moderate Exercise	47.20 (7.43)^a^	2.64 (1.47)	2.28 (2.84)
Intense Exercise	45.16 (9.87)	2.84 (1.40)	1.96 (2.17)

*^*a*^significantly different from rest.*

### Recognition Memory

The mean proportion of studied words, critical lures, and non-studied new words classed as *old* following each of the three activities, and the proportion of *remember*, *know*, and *guess* made responses to these words, are in Table 6. There was no significant multivariate effect of Condition on the proportion of words correctly recognized, λ = 0.86, *F*(1, 25) = 0.50, *p* = 0.80, η_*p*_^2^ = 0.14. Similarly, A MANOVA with Condition (Rest vs. Exercise) and Word Type (Studied vs. Critical Lure vs. New) as within-subjects factors, and judgment type (*Remember*, *Know*, *Guess*) as the dependent variables showed no significant multivariate effect of Condition on the proportion of words were recognized, λ = 0.82, *F*(6, 19) = 0.71, *p* = 0.65, η_*p*_^2^ = 0.18. As such, despite large proportions of studied words being accurately and critical lures falsely recognized, and few non-studied new words being recognized, exercise did not impact upon true and false recognition rates.

**TABLE 6:** Mean (SD) proportion of studied words correctly recognized, critical lures falsely recognized, and non-studied new items incorrectly recognized following each activity in Experiment 2.

		Rest	Moderate intensity exercise	High intensity exercise
Studied Words	Old (*Correct*)	0.84 (0.15)	0.85 (0.11)	0.84 (0.12)
	Remember	0.53 (0.25)	0.59 (0.59)	0.58 (0.24)
	Know	0.21 (0.24)	0.17 (0.19)	0.15 (0.22)
	Guess	0.10 (0.08)	0.09 (0.10)	0.10 (0.13)
Critical Lures	Old (*Incorrect*)	0.85 (0.18)	0.85 (0.21)	0.86 (0.22)
	Remember	0.52 (0.30)	0.59 (0.36)	0.53 (0.35)
	Know	0.24 (0.31)	0.15 (0.20)	0.24 (0.27)
	Guess	0.09 (0.14)	0.11 (0.17)	0.09 (0.18)
New Words	Old (*Incorrect*)	0.18 (0.24)	0.10 (0.24)	0.13 (0.13)
	Remember	0.03 (0.07)	0.02 (0.04)	0.05 (0.09)
	Know	0.01 (0.03)	0.03 (0.08)	0.02 (0.06)
	Guess	0.14 (0.23)	0.05 (0.09)	0.05 (0.08)

## General Discussion

The current study provides initial evidence that acute aerobic exercise can not only enhance free recall performance, but also that this benefit is not accompanied by changes in false recall. In Experiment 1, results demonstrated that moderate intensity exercise improved free recall performance compared to a rest condition, with no associated increase in false recall. In Experiment 2, moderate intensity exercise again improved memory performance, and that this benefit was also observed compared to an intense exercise condition. The intense exercise condition, however, did not result in an increase in false memory recall. This partially supports and advances the initial observations made by Loprinzi and colleagues (Dilley et al., 2019; Green and Loprinzi, 2018; Siddiqui and Loprinzi, 2018) that exercise may have beneficial effects on false memory.

By assessing both free recall and recognition memory, the data suggests that whilst free recall was benefited, recognition memory was not influenced by exercise. This supports the Dilley et al.’s (2019) findings and may be due to the different mechanisms underlying free recall and recognition (Diekelmann et al., 2010). For example, recognition tasks aid source monitoring processes through the reactivation of sensory details of the study words and their encoding context (Cabeza et al., 2001) and recognition decisions are based on inferential judgments. This can be evidenced by the similarly high levels of critical lure recognition in both experiments, but otherwise low levels in the recall test. Also, recall tests were completed before the recognition test, which has been shown to influence recognition rates (e.g., Roediger and McDermott, 1995). Similar temporal considerations are important for the impact of exercise, as the effects of exercise on self-reported arousal and blood lactate had all decreased by the recognition testing phase, reducing the effects of exercise on response bias. However, given that exercise induced arousal was elevated during encoding, this requires further consideration.

In line with FTT, these findings suggest that exercise did not influence gist based memory processes when compared to quiet rest. Concerning the increased correct recall of presented words, exercise appears to have increased verbatim processing through focusing active attention on the perceptual details of presented words (potentially through effective mental rehearsal). This suggestion is in line with the proposal that acute moderate intensity exercise facilitates attentional allocation and efficient information processing speeds during cognitive tasks (e.g., Hillman et al., 2003; Kamijo et al., 2007), also evidenced through larger P3 amplitude (Hillman et al., 2003; Kamijo et al., 2007) and shorter P3 latency (Hillman et al., 2003). This improved active attentional allocation supported the encoding of contextually specific information, and consequentially assisted the recall of presented words but not non-presented words that were merely semantically activated. It is also possible that post-exercise encoding may inhibit automatic spreading activation. Given that arousal and BL levels were highest immediately post-exercise, it is likely exercise biased the encoding phase. Indeed, researchers suggest acute exercise may primarily benefit encoding rather than consolidation (stabilization of memory traces post-encoding) (e.g., Labban and Etnier, 2011), although others highlight exercise benefiting consolidation (McNerney and Radvansky, 2015). These initial findings point to a limited impact of physical exercise on false memory generation.

One key limitation is that no exploration of long-term memory consolidation processes is possible, which other researchers have highlighted as a key role of exercise (e.g., Tomporowski and Pendleton, 2018). There is also the potential that as the effects of exercise remain long after exercise completion, retrieval processes may also have been influenced by exercise. In addition, the present study did not take into account baseline in memory performance. This may be an important consideration given that both propensity for false recall (e.g., Diekelmann et al., 2010) and influence of exercise on cognition (e.g., Sibley and Beilock, 2007) are sensitive cognitive capacity.

Whilst the present study addressed exercise intensity, the protocols in terms of intensity and manipulation were not without issue. For example, the American College of Sports Medicine (2013) considers moderate intensity exercise to be between 64–76% of estimated HRmax. In Experiment 1, heart rates were well within this range (68.40% HRmax), yet participants in Experiment 2 exercised on average at 79.43%HRmax in the equivalent condition. This suggests that intensity may have been higher than expected in Experiment 2, and across the sample 13 participants were above this HR range. Using perceptual data, only 6 participants rated their RPE as being above the 13 upper limit identified as indicating moderate intensity exercise. Importantly, significant differences across all conditions in Experiment 2 for RPE and HR suggest that the conditions were distinct in terms of intensity. Yet a consequence is that across the experiments, moderate intensity exercise conditions were different not only in terms of intensity, but also the resultant affective experience. With Experiment 1 participants experienced moderate intensity exercise (RPE between somewhat hard and hard, with option to adjust intensity) as more positive than in Experiment 2 (intensity set at 60% of VO2peak). This may have been a result of the self-controlled nature versus prescriptive protocols employed, with self-selected exercise associated with more positive affective responses through perceived autonomy (Oliveira et al., 2015). Future research should explore the role of exercise intensity and its manipulation in terms of true and false memory generation. Finally, the generalizability of these findings are limited by the use of regularly physically active samples of young adults. Whilst there is limited evidence on the moderating role of fitness in the acute exercise and memory relationship (Loprinzi et al., 2019), fitness status is acknowledged an important consideration (Chang et al., 2012). Therefore, future studies are needed to explore the roles of exercise intensity and fitness, as well as other individual differences (e.g., cognitive capacity, age), proposed to moderate acute exercise and cognitive performance relationship.

In conclusion, the present study found that acute bouts of moderate intensity aerobic exercise performed before encoding and immediate retrieval of semantically associated words improved the volume of studied information correctly recalled. There were no associated increases in false memory generation, suggesting that exercise induced arousal facilitated verbatim memory traces rather than promoting gist-based processing at encoding. This is in line with Lambourne and Tomporowski (2010) meta-regression analysis observations that post-exercise exercise-induced arousal facilitates speeded mental process, as well as enhancing memory storage and retrieval, even if the exercise was intended to induce physical fatigue. This also supports initial proposals made by Loprinzi and colleagues (Green and Loprinzi, 2018; Siddiqui and Loprinzi, 2018; Dilley et al., 2019), and the encoding acute exercise benefits suggested by Labban and Etnier (2018). There may be a limitation to this arousal effect, as intense exercise did not result in improvements in free recall performance, potentially due to more negative affect during intense exercise compared to moderate intensity exercise. The beneficial effects may be associated with acute exercise’s support of attentional processes and allocation, as well as arousal increasing verbatim processing of information. Research is required to address the underlying mechanisms and exercise characteristics (i.e., intensity, mode, duration and timing) that influence these false memory effects.

## Data Availability Statement

The raw data supporting the conclusions of this article will be made available by the authors, without undue reservation.

## Ethics Statement

The studies involving human participants were reviewed and approved by Edge Hill University Department of Sport and Physical Activity Ethics Committee. The participants provided their written informed consent to participate in this study.

## Author Contributions

DM, CT, and KM conceived, designed, and prepared the materials. DM and KM supervised the data collection. SH and LF collected the data. DM wrote the first draft. DM, CT, KM, SH, and LF contributed to the final approval of the version to be published and agreed to be accountable for all aspects of the work. All authors contributed to revising drafts critically for important intellectual content.

## Conflict of Interest

The authors declare that the research was conducted in the absence of any commercial or financial relationships that could be construed as a potential conflict of interest.
